# The Chinese Society of Clinical Oncology (CSCO): clinical guidelines for the diagnosis and treatment of gastric cancer

**DOI:** 10.1186/s40880-019-0349-9

**Published:** 2019-03-18

**Authors:** Feng-Hua Wang, Lin Shen, Jin Li, Zhi-Wei Zhou, Han Liang, Xiao-Tian Zhang, Lei Tang, Yan Xin, Jing Jin, Yu-Jing Zhang, Xiang-Lin Yuan, Tian-Shu Liu, Guo-Xin Li, Qi Wu, Hui-Mian Xu, Jia-Fu Ji, Yuan-Fang Li, Xin Wang, Shan Yu, Hao Liu, Wen-Long Guan, Rui-Hua Xu

**Affiliations:** 1 Department of Medical Oncology, Sun Yat-sen University Cancer Center, State Key Laboratory of Oncology in South China, Collaborative Innovation Center for Cancer Medicine, Guangzhou, 510060 Guangdong P. R. China; 2 Department of Gastrointestinal Oncology, Key Laboratory of Carcinogenesis and Translational Research (Ministry of Education), Department of Gastrointestinal Oncology, Peking University Cancer Hospital and Institute, Beijing, 100142 P. R. China; 3 Department of Oncology, Shanghai East Hospital, Tongji University School of Medicine, Shanghai, 200120 P. R. China; 4 Department of Gastric Surgery, Sun Yat-sen University Cancer Center, State Key Laboratory of Oncology in South China, Collaborative Innovation Center for Cancer Medicine, Guangzhou, 510060 Guangdong P. R. China; 5 Department of Gastric Cancer, Tianjin Medical University Cancer Institute & Hospital, National Clinical Research Center of Cancer, Tianjin’s Clinical Research Cancer for Cancer, Key Laboratory of Cancer Prevention and Therapy, Tianjin, 300060 P. R. China; 6 Medical Imaging Department, Key Laboratory of Carcinogenesis and Translational Research (Ministry of Education), Department of Gastrointestinal Oncology, Peking University Cancer Hospital and Institute, Beijing, 100142 P. R. China; 7 Pathology Laboratory of Gastrointestinal Tumor, The First Hospital of China Medical University, Shenyang, 110001 Liaoning P. R. China; 8 Department of Radiation Oncology, National Cancer Center, China and Cancer Hospital, Chinese Academy of Medical Sciences and Peking Union Medical College, Beijing, 100021 P. R. China; 9 Department of Radiotherapy, Sun Yat-sen University Cancer Center, State Key Laboratory of Oncology in South China, Collaborative Innovation Center for Cancer Medicine, Guangzhou, 510060 Guangdong P. R. China; 10 Department of Medical Oncology, Tongji Hospital Affiliated to Tongji Medical College of Huazhong University of Science and Technology, Wuhan, 430030 Hubei P. R. China; 11 Department of Medical Oncology, Zhongshan Hospital Affiliated to Fudan University, Shanghai, 200032 P. R. China; 12 Department of General Surgery, Nanfang Hospital Affiliated to Southern Medical University, Guangzhou, 510515 Guangdong P. R. China; 13 Department of Endoscopy Center, Key Laboratory of Carcinogenesis and Translational Research (Ministry of Education), Department of Gastrointestinal Oncology, Peking University Cancer Hospital and Institute, Beijing, 100142 P. R. China; 14 Department of Surgical Oncology, The First Hospital of China Medical University, Shenyang, 110001 Liaoning P. R. China

**Keywords:** Chinese Society of Clinical Oncology (CSCO), Gastric cancer, Diagnosis, Surgery, Neoadjuvant, Adjuvant, Radiotherapy, Chemotherapy, Targeted therapy, Immunotherapy

## Abstract

China is one of the countries with the highest incidence of gastric cancer. There are differences in epidemiological characteristics, clinicopathological features, tumor biological characteristics, treatment patterns, and drug selection between gastric cancer patients from the Eastern and Western countries. Non-Chinese guidelines cannot specifically reflect the diagnosis and treatment characteristics for the Chinese gastric cancer patients. The Chinese Society of Clinical Oncology (CSCO) arranged for a panel of senior experts specializing in all sub-specialties of gastric cancer to compile, discuss, and revise the guidelines on the diagnosis and treatment of gastric cancer based on the findings of evidence-based medicine in China and abroad. By referring to the opinions of industry experts, taking into account of regional differences, giving full consideration to the accessibility of diagnosis and treatment resources, these experts have conducted experts’ consensus judgement on relevant evidence and made various grades of recommendations for the clinical diagnosis and treatment of gastric cancer to reflect the value of cancer treatment and meeting health economic indexes. This guideline uses tables and is complemented by explanatory and descriptive notes covering the diagnosis, comprehensive treatment, and follow-up visits for gastric cancer.

## Background

Gastric cancer is a common type of malignant tumor with relatively poor prognosis and presents a serious threat to global health. According to the International Agency for Research on Cancer (IARC), in 2012, there were approximately 951,000 newly diagnosed cases of gastric cancer worldwide and 723,000 related deaths. Among all cancers, gastric cancer ranks fifth in terms of incidence and third in terms of mortality worldwide [1].

More than 70% of new gastric cancer cases are found in developing countries. About 50% of these cases are in East Asia, in which China is the most affected country as it accounts for 42.6% of the global incidence and 45% of all gastric cancer-related deaths [1, 2]. In February 2018, the latest statistics from the Chinese National Cancer Center showed that although the overall incidence of gastric cancer is declining, it still remains second in terms of incidence among all malignancies in China, just below lung cancer [3, 4]. In terms of incidence, it is ranked second and fifth among males and females, respectively. In terms of mortality, it is ranked third, preceded by lung cancer and liver cancer, whereby the mortality among males and females are ranked third and second, respectively [3].

The common risk factors of gastric cancer include *Helicobacter pylori* (HP) infection, smoking, high salty-diets, susceptibility to hereditary gastric cancer syndrome. However, since it has a complex microenvironment and is a heterogeneous disease, there exist differences between the Western and Eastern gastric cancer populations as to the etiology, epidemiological characteristics, primary tumor site, histopathology, treatment strategies, prognoses, molecular biological characteristics, and immunological characteristics. The incidence of proximal gastric cancer is rising in the West, and that of non-proximal locations are rising in the East, especially in Japan and China. In the East, gastric cancer is often diagnosed in its early stages in countries like Japan and Korea, but, in China more than 80% of gastric cancer patients are already in advanced stages at the time of diagnosis; for which many may miss the opportunity of radical resection or may have high risks of postoperative metastasis and relapse. In addition, there are differences in the treatment practices among different regions in China. As such, the purpose of this guideline is to standardize the treatment for the different stages of gastric cancer in the Chinese population.

## Diagnosis

### Basic principles

The tumor-node-metastasis (TNM) staging system from the American Joint Cancer Committee/Union Internationale Contre le Cancer (AJCC/UICC) is the internationally accepted standard for gastric cancer staging, and the 8th edition is used throughout this guideline. Initial evaluation of gastric cancer mainly includes imaging and pathological examinations for qualitative, location, and staging diagnosis. Other examinations include complete physical examination, blood chemistry tests, endoscopy (endoscopic ultrasound [EUS] and fine-needle biopsy), metastatic lesion biopsy, diagnostic laparoscopy, and diagnostic intra-peritoneal fluid examination. Histopathological examination is the gold-standard for gastric cancer diagnosis and a basic requirement prior to treatment initiation. Thoracic, abdominal, and pelvic computed tomography (CT) is the primary diagnostic modality used for pre-treatment clinical staging. Magnetic resonance imaging (MRI), laparoscopic exploration, and positron emission tomography (PET) scan are alternatives to CT for the diagnosis of liver, peritoneal, and systemic metastases. The imaging report should describe observations to support the clinical stage evaluation and classification (cTNM). The postoperative histopathological diagnosis (pTNM) should provide information for identifying the histological subtype of the tumor and full assessment of the tumor (including location, lymph node status, and the number of lymph nodes retrieved), which are important for prognostication and planification of personalized treatment strategies. At present, the molecular classification of gastric cancer is based on the human epidermal growth factor receptor 2 (HER2) expression in the tumoral tissue, and it is the basis for selecting anti-HER2 targeted therapy. All cases pathologically diagnosed as gastric or esophagogastric junction (EGJ) adenocarcinoma should undergo HER2 assessment.

### Imaging and endoscopy

**Table:** 

Purpose (diagnosis/evaluation)	Grade I recommendations	Grade II recommendations	Grade III recommendations
Qualitative	Gastroscopy + biopsy (Evidence 1A)	Cytological examination (Evidence 2A)^a^	
Location	Gastroscopy (Evidence 1A) Abdominal enhanced CT scan (Evidence 1A)	Abdominal MRI (Evidence 2A)	X-ray barium double contrast radiography (Evidence 2B)
Staging	Abdominal and pelvic enhanced CT scan^b^ (Evidence 1B) Chest CT^c^ (Evidence 1B) EUS^d^ (Evidence 1A)	Abdominal MRI^e^ (Evidence 2A) PET/CT (Evidence 2A) Diagnostic laparoscopy and examination of intraperitoneal washings^f^ (Evidence 1B)	
Treatment efficacy	Abdominal and pelvic enhanced CT scan^g^ (Evidence 1A)	Gastroscopy (Evidence 2A) PET/CT (Evidence 1B) Abdominal MRI (Evidence 2A)	Functional imaging examination^h^ (Evidence 3)

**Notes**

- When repeated gastroscopic biopsies are unable to confirm the pathological diagnosis, cytological examination of the ascites/pleural effusion or the pathological examination of the metastatic lesions can be used as the basis for qualitative diagnosis.
- Ensure that the gastric cavity is fully dilated and expanded by drinking 500 mL of liquid, water preferably, prior to the examination [5]. A multiphase and multi-planar enhanced contrast scan should be used for diagnosis. Plain abdominal CT scans are not recommended. If patients have contraindications to the contrast agent used for enhanced CT scan, MRI or EUS is recommended.
- Chest CT can detect and show lung metastasis better than X-rays [6]. For carcinoma of the EGJ, an enhanced CT scan of the chest should be performed to judge the range and metastatic status of mediastinal lymph nodes.
- EUS should be carried out in qualified centers only. In the 8th edition of the AJCC/UICC staging system for gastric cancer [7], esophageal cancer, and EGJ cancer, EUS is recommended as the preferred cT staging modality for the clinical evaluation of the depth of tumor invasion; EUS cT staging not only enables direct observation of the lesions but can also provide visual descriptions about the different anatomical layers of the gastric wall. The tumor is mostly manifested in the non-homogeneous hypoechoic region, which is usually accompanied by the destruction of the corresponding layers of the gastric wall. Simultaneously, EUS can detect enlarged perigastric lymph nodes and metastatic lesions in the gastric-neighboring parts of the liver and peritoneal cavity. In all, EUS is helpful for the diagnosis, clinical staging, and assessment of response of gastric cancer to neoadjuvant therapy. Systematic analysis has identified the overall sensitivity and specificity of EUS in distinguishing between T1/2 and T3/4 cancers as 0.86 and 0.90, between T1 and T2 cancers as 0.85 and 0.90, and between T1a and T1b cancers as 0.87 and 0.75, respectively [8].
- When liver metastasis is suspected on a CT scan, abdominal MRI is recommended for further confirmation [9]. If the patients’ conditions permit, a hepatocyte-specific contrast agent can be used to increase the diagnostic sensitivity.
- Diagnostic laparoscopic exploration and examination of intraperitoneal washings are recommended for detecting occult metastasis and when peritoneal metastasis is suspected [7]. For intraperitoneal lavage, 200 mL of normal saline can be infused into the different quadrants of the abdominal cavity, and the surgeons can collect more than 50 mL of the lavage fluid for cytological examinations.
- According to the RECIST 1.1 guidelines [10], the nodules of the liver, lung, or peritoneal metastasis with a long-axis diameter ≥ 1 cm or lymph nodes with a short-axis diameter ≥ 1.5 cm should be used as target lesions for treatment evaluation. The thickness of primary lesions can be used as a reference for therapeutic assessment but should not be considered as target lesion.
- Small-scale studies have demonstrated that functional imaging parameters such as the apparent diffusion coefficient value of diffusion MRI [11] and iodine concentration of energy spectral CT [12] can assist in evaluating the curative effect of gastric cancer therapies and can be used as reference indexes for evaluation in atypical cases.

### Pathological diagnosis

#### Histopathological diagnosis

**Table:** 

Sample Type	Grade I recommendations		Grade II recommendations	Grade III recommendations
	Gross examination	Light microscopic examination		
Biopsy specimen*	Record the size and number of tissues biopsied	Identify the nature and histological type of the lesion Cancerous/non-cancerous Benign/malignant Histological subtype	Detect immunohistochemical markers^m^: used for differential diagnosis of histological subtypes, confirmation of vascular and lymphatic invasion, evaluation of tumor cell proliferation activity, etc	Evaluate the presence of HP infection^n^ (Evidence 1B)
Endoscopic resection specimen^a^ (EMR/ESD)	Tumor site^b^ Tumor size (cm^3^)	Intra-epithelial neoplasia/adenomatous grade (high grade) Invasive carcinoma Histological subtype^d^/Lauren classification^e^ Histological grade (G1, G2, G3) Depth of infiltration Horizontal distal margin and deepest infiltration margin Vascular and lymphatic invasion	Same as above The general type of early-stage gastric cancer^k^	Same as above
Surgical resection specimens for those without neoadjuvant therapy	Type of the surgical specimen Tumor site Tumor size (cm^3^) The distance of the tumor lesion from the lateral edge of the mouth/anus The stations and number and of lymph nodes retrieved (At least 16 lymph nodes and/or preferentially > 30 lymph nodes to be retrieved)^c^	Histological subtype/Lauren classification Histological grade (G1, G2, G3) Depth of invasion (pT classification)^f^ Vascular, lymphatic, and nerve invasion Margin lateral to the mouth/anus^g^ Invasion to the esophagus/duodenum (if resected) Number of lymph node metastases/number of lymph nodes retrieved (pN classification) Number of cancer nodules^h^ Distant metastasis (pM stage)^i^ pTNM staging of gastric cancer (8th AJCC/UICC edition)^b^	Same as above General type of progressive gastric cancer^l^	Same as above
Surgical resection specimens for those who had neoadjuvant therapy	Same as above (for specimens with no obvious tumors, careful examination and multipoint sampling should be made to avoid misdiagnosis of response to tumor therapy and clinicopathological stage)	Same as above Tumor regression grade (TRG)^j^ ypTNM stage (8th AJCC/UICC edition)	Same as above	Same as above

* For non-resectable lesions, the cytological assessment of ascites and pleural effusion and biopsy of the distant metastatic lesion should be conducted as routinely performed

**Notes**

- Endoscopic mucosal resection (EMR)/endoscopic sub-mucosal dissection (ESD) has become the new alternative treatment of early-stage gastric cancer [13]. EMR/ESD specimens should be meticulously resected, collected, and prepared based on standard protocols [14].
- According to the 8th edition of the AJCC/UICC staging system for gastric cancer [7], esophageal cancer, and EGJ carcinoma, the staging criteria for EGJ carcinoma or gastric-cardia carcinoma are defined as follows: (1) if the tumor invades the gastroesophageal boundary and the tumor’s epicenter is within 2 cm from the EGJ, staging criteria for esophageal cancer should be adopted; (2) if the tumor invades the gastroesophageal boundary but its epicenter is located > 2 cm from the EGJ, the staging criteria for gastric cancer should be adopted. It is worth noting, that among the total number of gastric cancer cases analyzed by the AJCC/UICC committee for providing such definition, the number of Chinese cases analyzed were very limited and may be a source for potential bias [15]. The data from a single-center study regarding Chinese patients with gastric-cardia cancer showed that the biological behavior and clinical characteristics of EGJ carcinoma (Siewert II cardiac cancer) had greater similarities to gastric cancer than to esophageal cancer [16]. However, studies comprising of larger cohort of patients are required for validation.
- To avoid inaccuracy for staging tumors according to the AJCC/UICC pTNM staging system, ≥ 16 lymph nodes should be pathologically evaluated. For a more accurate evaluation, the preferred number of lymph nodes should be > 30 [7]. In order to help clinicians to accurately determine the range of lymph node metastasis, it is recommended that surgeons and pathologists to collect and group the perigastric lymph nodes according to their respective stations, which should be accordingly mentioned in the postoperative pathological report in addition to providing the total number of metastatic lymph nodes and total number of lymph nodes examined. For instance, this can be based on the following example:
  Lymph node station no. χ: number of metastatic lymph nodes/number of examined lymph nodes.
- When the pathological diagnosis is difficult, the specimens should be sent to a more specialized center/hospital for further evaluation. To enable a proper assessment of the specimen, the items to be sent should include (1) the original pathological report (for cross-checking analysis with the pathological slides), (2) sufficient pathological glass slides and/or paraffin blocks, and (3) the detailed surgical records.
- According to the Lauren classification [17], gastric adenocarcinoma is classified as intestinal type, diffuse type, and mixed type based on its histological growth patterns.
- According to the 8th edition of the AJCC/UICC staging system [7], gastric cancer invading the muscularis propria is to be classified as “T2”, but there is no detailed classification for tumors invading into the superficial muscularis propria layer and deeper muscularis propria. Based on the results of a large-cohort domestic single-center study [18], the prognoses of patients with tumor invading the deeper sub-mucosal layer were significantly worse than those of patients with tumor invading the superficial sub-mucosal layer. Therefore, when the tumor invasion is limited to the muscularis propria, specific mentions as to whether the tumor has invaded the superficial or deep sub-mucosal layer should be recorded in the final pathological report. This will be helpful in evaluating the patient’s prognosis and for the planification of personalized treatment strategies.
- This guideline defines a positive surgical margin as the presence of cancer cells within a 1 mm distance from the resected margin.
- The detection of carcinomatous nodules in sub-serous adipose tissues adjacent to the primary tumor site is to be considered as regional lymph node metastasis even if there is no evidence of residual lymph node tissues [7]. It is recommended that regional metastatic lymph nodes and carcinomatous nodules are to be recorded separately.
- If tissues obtained from non-neighboring regions of the stomach is pathologically confirmed as being metastatic, these are to be regarded as distant metastasis (pM1), which includes metastatic tissues from distant lymph node stations and cancerous cells detected in other organs (including intraperitoneal washings or peritoneal seedings). Gross evidence of metastasis seen during surgery can be recorded in the final pathological report as distant metastasis (cM) and can be reported as pTpNcM0-1.
- The classification, description, and evaluation proposals from the National Comprehensive Cancer Network (NCCN) Clinical Practice Guidelines for Gastric Cancer [19], the AJCC/UICC pTNM staging system [7], and the latest version of the Japanese Gastric Cancer Treatment Guidelines has been well accepted by Chinese pathologists as they have been observed to be highly applicable in Chinese patients, particularly in relation to the grading of tumor regression based on the degree of tumor cell residues and fibrosis. Of note, the pathological staging of gastric cancer after neoadjuvant therapy from the 8th AJCC/UICC staging system has fully considered the limitations of existing assessment methods and their association with prognosis and has simplified the ypTNM classification to a certain extent.
- Early-stage gastric cancer is defined as gastric cancer confined to the mucosa and submucosa, regardless of whether there is evidence of regional lymph node metastasis.
- Advanced gastric cancer is defined as a tumor which has invaded the muscularis propria or deeper layer of the gastric wall. The Borrmann classification includes four subtypes: Borrmann Type I, nodular polypoid tumor; Type II, local ulcerative tumor with easily identified margin; Type III, infiltrating ulcerative tumor with poorly defined margins; and Type IV, poorly demarcated, infiltrative, and diffuse tumor (local Borrmann Type IV, gastric tumor infiltrating the linitis plastica).
- When the pathological diagnosis is difficult to determine, gastric cancer-related markers can be used for confirming the diagnosis, differential diagnosis, prognostic evaluation, and follow-up/treatment needs [20].
- The 8th edition of the AJCC/UICC staging system for gastric cancer requires the recording of the HP infection status and thus, it should be assessed and recorded in medical institutions having such facilities [21].

#### Molecular classification

**Table:** 

Molecular classification	Grade I recommendations	Grade III recommendations
After a pathological diagnosis of gastric cancer, molecular profiling should be conducted and treatment should be guided according to the molecular classification	All cases of gastric adenocarcinoma should undergo HER2 assessment^a–d^ (Evidence 1A)	
Molecular profiling related to prognosis of gastric cancer		HER2 assessment^e,f^ (Evidence 3) MSI/MMR detection^g,h^ (Evidence 3)

**Notes**

- HER2-positive tumor is a unique subtype of gastric cancer, and its diagnostic and treatment modalities are different from HER2-negative gastric cancer [22].
- HER2-positive late-stage gastric cancer patients may have survival benefits from trastuzumab therapy [23]. The level of HER2 gene amplification can be used to predict the sensitivity to trastuzumab therapy and potential survival benefits for these patients [23–27].
- For primary lesions after neoadjuvant therapy as well as recurrent and/or metastatic lesions, if sufficient specimens can be obtained, it is recommended to re-assess the HER2 amplification level [28].
- Both gastroscopic biopsy specimens and surgical specimens can be used for HER2 assessment [29].
- Immunohistochemistry (IHC) and in situ hybridization (ISH) for HER2 assessment should be strictly performed in accordance with the Guidelines for HER2 detection in gastric cancer (2016 edition) [30]. The related tests (IHC, FISH/double signal in situ hybridization [DSISH]) should be performed using the China Food and Drug Administration (CFDA) approved kits.
- The positive rate of HER2 overexpression in gastric cancer reported worldwide is between 7.3 and 20.2%, but in Chinese gastric cancer patients, it ranges between 7 and 12% [31, 32]. The results of retrospective studies have shown that HER2 positive expression was associated with old age, males, intestinal type, and tumors located in the upper third of the stomach [24, 33]. Different studies have used different HER2 evaluation criteria. As a result, there is no consensus concerning the prognostic/predictive value of HER2 for gastric cancer. Studies have shown that HER2 was associated with poor prognosis in early-stage gastric cancer patients and was not an independent prognostic factor for advanced gastric cancer patients [34, 35]. A retrospective study comprising of 838 gastric cancer patients from all stages demonstrated that the best survival outcomes were observed for patients with HER2-negative intestinal-type gastric cancer, whereas the worst survival outcomes were for those with HER2-positive diffuse-type gastric cancer [33].
- In a meta-analysis [36] comprising of 8 studies (1976 cases), the rate of high microsatellite instability (MSI-H) was between 11.68 and 33.82%. Of the four studies which used the National Cancer Institute (NCI) standards to define MSI-H, three studies suggested that MSI-H was more likely to be found in intestinal-type gastric cancer and was related with a better prognosis. It was reported that MSI-H/mismatch repair deficient (dMMR) patients who underwent preoperative chemotherapy followed by surgery had a poorer prognosis as compared to those who underwent surgery only [37]. Therefore, MSI/MMR status may help to screen gastric cancer patients favorable for preoperative chemotherapy.
- The most commonly used method to detect MMR expression status is the detection of MMR-related proteins with IHC and the detection of multiple microsatellite instable (MSI) loci with polymerase chain reaction (PCR). Simple and economical IHC for MMR proteins is easily carried out in pathological laboratories. At present, commercialized specific monoclonal antibodies against four MMR proteins (MLH1, PMS2, MSH2, and MSH6) are available in China. IHC detection is carried out in pathological departments of many large tertiary hospitals. For institutions with adequate facilities, PCR-capillary electrophoresis can be used to detect MSI loci.

## Comprehensive treatment of gastric cancer

### Treatment of non-metastatic gastric cancer

#### Treatment of resectable gastric cancer

The treatment of resectable gastric cancer is based on the evaluated clinical stage. The primary choice of treatment for early-stage gastric cancer is endoscopic treatment, which includes EMR or ESD. For patients unsuitable for endoscopic treatment, laparotomy or laparoscopy can be performed. Patients identified as having metastatic lymph nodes, confirmed by postoperative pathology, should undergo postoperative chemotherapy. The standard treatment for locally advanced gastric cancer is D2 gastrectomy followed by postoperative adjuvant chemotherapy. Neoadjuvant therapy is also among one of the recommendations for advanced resectable gastric cancer patients (clinical stage III or above). However, for patients with progressive disease unable to undergo R0 resection after neoadjuvant treatment, till present, there is no adequate evidence-based data to support remedial therapy, but the best treatment plan can be formulated through a multidisciplinary team (MDT) discussion based on the individual’s condition. Radiochemotherapy is an alternative choice for patients with resectable tumors but unsuitable for surgery due to individual factors. However, personalized treatment should be tailored so as to provide an optimal treatment strategy.

##### Endoscopic therapy for early-stage gastric cancer

**Table:** 

Stage	Stratification	Grade I recommendations	Grade II recommendations
cT1aN0M0, Stage I	Patients suitable for EMR/ESD^a^	Patients who had non-radical resection with EMR/ESD must be re-operated (Evidence 1A)^b^	Patients with non-radical resection must receive additional ESD, electrotomy, or close follow-up upon providing informed consent (Evidence 2A)^c–f^

**Notes**

- For some early-stage gastric cancer patients, endoscopic therapy (EMR/ESD) can be used as an alternative to conventional surgery [38, 39].
- EMR/ESD should be performed in experienced medical centers only [40]. The absolute indications for endoscopic therapy as recommended by the Expert Committee are (1) intra-mucosal carcinoma < 2 cm and visible to the naked eyes (cT1a); (2) well-differentiated carcinoma (papillary adenocarcinoma, well-differentiated tubular adenocarcinoma, and moderately differentiated tubular adenocarcinoma); (3) regardless of the general type of the tumor, resection should be limited to non-ulcerative lesion. Indications for extended endoscopic therapy are (1) non-ulcerative and well-differentiated cT1a tumors > 2 cm; (2) ulcerative and differentiated cT1a tumors < 3 cm; and (3) non-ulcerative and undifferentiated cT1a tumor < 2 cm, for which vascular invasion is absent and the probability for potential lymph node metastasis is low [41]. For larger lesions which have a high probability of incomplete resection by EMR, ESD is recommended [42].
- Some Chinese scholars are exploring the efficacy of ESD in T1b patients, for which the incidence rate of lymph node metastasis is relatively low (about 15%–25%). As such, T1b patients who are unsuitable for surgery or chemoradiotherapy due to old age or underlying disease conditions can be enrolled and treated in clinical trials at specialized institutions.
- Indications for radical surgery are complete resection of all lesions, tumor diameter < 2 cm, well-differentiated tumor, invasion depth characterized as pT1a, non-ulcerative lesions, negative surgical margin, and absence of lymphatic and vascular invasion [41].
- Indications for extended radical resection are that the completely resected specimen should meet the following criteria: (1) non-ulcerative lesion > 2 cm, well-differentiated pT1a tumor; (2) ulcerative lesion < 3 cm, well-differentiated pT1a tumor; (3) non-ulcerative lesion < 2 cm, undifferentiated pT1a tumor; (4) well-differentiated lesions < 3 cm, classified as pT1b-SM1 (< 500 μm from the muscularis mucosae), having negative resection margin and without lymphatic and vascular invasion [42].
- Non-radical resection refers to tumors that fail to meet any of the indications for radical or extended radical resection and should undergo additional elective surgery. However, for tumors considered as being non-radically resected mainly because the tumor could not be removed as a single piece or for those which are completely resected but have positive horizontal margins, additional surgery resection may not be the only choice. According to the current diagnosis and treatment principles, these patients can undergo additional ESD, electric resection, or continuous close follow-up visits after providing signed consent forms [43].

##### Surgical treatment of resectable gastric cancer

*3.1.1.2.1 Overall treatment strategy*

**Table:** 

Clinical staging*	Stratification	Grade I recommendations	Grade II recommendations	Grade III recommendations
Stage I				
cT1aN0M0	Patients unsuitable for EMR/ESD	D1 gastrectomy (Evidence 1A)	Laparoscopic D1 gastrectomy (Evidence 1B)	
cT1bN0M0	Patients suitable for surgery	D1 gastrectomy (differentiated type, < 1.5 cm) or D1 + gastrectomy (undifferentiated type, < 1.5 cm) (Evidence 1A)	Laparoscopic D1/D1 + gastrectomy (Evidence 1B)	
cT2N0M0	Patients suitable for surgery	D2 gastrectomy (Evidence 1A)	Laparoscopic D2 gastrectomy (Evidence 2A)	
Stage II				
cT1-2N1-3M0, cT3-4N0M0	Patients suitable for surgery	D2 gastrectomy + adjuvant chemotherapy (Evidence 1A)	Laparoscopic D2 gastrectomy (Evidence 2A) + adjuvant chemotherapy (Evidence 1)	
Stage III				
cT3-4aN1-3M0	Patients suitable for surgery	D2 gastrectomy + adjuvant chemotherapy (Evidence 1A)	Laparoscopic exploration (Evidence 2B) Neoadjuvant chemotherapy + D2 gastrectomy + adjuvant chemotherapy (Evidence 2A) EGJ carcinoma: neoadjuvant chemoradiotherapy + D2 gastrectomy + adjuvant chemotherapy (Evidence 1B)	D2 gastrectomy + adjuvant chemoradiotherapy (Evidence 3)
Stage IVA				
cT4bN0-3M0	No unresectable factors**	MDT discussion for the optimal treatment regimen	Participation in clinical trials should be encouraged	
Stage I–IVA	Patients unsuitable for surgery	See “Comprehensive Treatment of Unresectable Gastric Cancer” for the principles of treatment		

* The 8th edition of the AJCC/UICC clinical staging system (cTNM)

** Unresectable factors are (1) tumors with involvement of the mesenteric root or para-aortic lymph nodes (highly suspected on imaging or confirmed by biopsy), (2) tumors have invaded or encapsulated important surrounding blood vessels (excluding the splenic artery), and (3) distant metastasis or peritoneal seeding (including positive cytological examination of intraperitoneal washings) [19]

*3.1.1.2.2 Principles of surgery*

**Table:** 

Technical requirement	Type of gastrectomy	Grade I recommendations	Grade II recommendations	Grade III recommendations
Type of lymphadenectomy	Distal gastrectomy			
	D1	Lymph node stations: No. 1, 3, 4sb, 4d, 5, 6, 7		
	D1+	Lymph node stations: D1 + No. 8a, 9		
	D2	Lymph node stations: D1 + No. 8a, 9, 11p, 12a (Evidence 1A)	Selective resection of lymph node 14v* based on D2 lymphadenectomy (Evidence 2A)	
	Proximal gastrectomy			
	D1	Lymph node stations: No. 1, 2, 3a, 4sa, 4sb, 7 (Evidence 1A)		
	D1+	Lymph node stations: D1 + No. 8a, 9, 11p (Evidence 1A)**		
	D2			Lymph node stations: D1 + No. 3b, 8a, 9, 11p
	Total gastrectomy			
	D1	Lymph node stations: No. 1–7		
	D1+	Lymph node stations: D1 + No. 8a, 9, 11p (Evidence 1A)**		
	D2	Lymph node stations: No. 1 to 7, 8a, 9, 10, 11, 12a (If the tumor has invaded the esophagus, stations No. 19, 20, 110, and 111 should be dissected) (Evidence 1A)	Similar as Grade I D2 stations + station No. 10*** (Evidence 2A)	
Digestive tract reconstruction	Distal gastrectomy	Billroth I (Evidence 1A) Billroth II (Evidence 1A)		Roux-en-Y anastomosis (Evidence 2B)
	Proximal gastrectomy	Esophagogastrostomy (Evidence 1A)	Tubular gastroesophageal anastomosis (Evidence 2A)	Jejunal interposition for gastric replacement (Evidence 2B)
	Total gastrectomy	Roux-en-Y anastomosis (Evidence 1A)		Roux-en-Y anastomoses with jejunal pouch reconstruction (Evidence 2B) Jejunal interposition for gastric replacement (Evidence 2B)

* For patients with metastatic lymph nodes in the middle and lower portions of the stomach, and preoperative stage evaluation considered as cT3 or cT4

** Proximal gastrectomy D1+ is recommended for early-stage proximal gastric cancer

*** For patients with primary tumor > 6 cm, located at the greater curvature, in the upper or middle portions of the stomach, and preoperative stage evaluation considered as cT3 or cT4

**Notes**

*Principles of surgery*

- The scope of gastrectomy is based on the location of the tumor, with an aim to ensure adequate surgical resection margin. In the past, a surgical margin of ≥ 4 cm from the tumor was recommended. Based on data from recent studies [41], the recommendations for an adequate distance of resection margin for Borrmann I–II gastric cancers are to be ≥ 3 cm, and for Borrmann III–IV it should be ≥ 5 cm. If the tumor has invaded the esophagus or pylorus, a resection margin of 5 cm is not obligatory only when R0 resection can be assured and the frozen pathological examinations of the resection margins are negative.
- For EGJ adenocarcinoma which has invaded < 3 cm into the esophagus or the body of the stomach, non-endoscopic surgery is recommended. Transthoracic surgery is not recommended because of the risks of complications and mortality are significantly increased, without significant improvement in survival [44].
- The resection of perigastric lymph nodes and those alongside accompanying vessels of the abdominal cavity should be performed according to the type of gastrectomy. D1 gastrectomy includes the resection of the required part of the stomach (with adequate resection margin), greater and lesser omentum, and the following perigastric lymph nodes: the right and left para-cardial lymph nodes, lesser and greater curvature lymph nodes, lymph nodes along the left gastric artery, suprapyloric, and infrapyloric lymph nodes along the right gastric artery. D2 gastrectomy includes the structures resected in D1 gastrectomy and, in addition, the resection of the lymph nodes along the common hepatic artery, celiac artery, splenic hilum, and splenic artery. D2 lymph node dissection is the standard recommendation for resectable gastric cancer classified as being cT1N+ and cT2-4N-/+. It is recommended that ≥ 16 lymph nodes should be pathologically examined to ensure accurate staging and prognostication [45]. It is worth mentioning that proximal gastrectomy is recommended for early-stage patients (T1–2N0), and in the 4th edition of the Japanese Gastric Cancer Treatment Guidelines [41], D1 or D1+ lymphadenectomy is recommended for this group of patients. However, after a study has demonstrated that the rates of lymph node metastasis for EGJ adenocarcinoma having diameter < 4 cm (the majority was Siewert II tumors) at stations 4sa, 4sb, 4d, 5, 6 were less than 1% [46], in the latest edition of the Japanese Gastric Cancer Treatment Guidelines, the recommended scope of D2 proximal gastrectomy was revised to D1 resection including lymph node stations 3b, 8a, 9, and 11p.
- There is great controversy over the necessity for splenic hilar lymph node dissection. The rate of splenic hilar lymph node metastasis varies greatly in different reports [47–51]. At present, it is recommended that splenic hilar lymph node dissection should not be performed for patients with stage cT1–2 gastric cancer [52]. The Expert Committee recommends that splenic hilar lymph node dissection should be performed in the following cases: the primary tumor is > 6 cm, located at the greater curvature and middle-upper part of the stomach, and preoperatively staged as T3–4. Splenectomy for the purpose of lymph node dissection is not recommended [53].
- Whether it is necessary to dissect lymph nodes at the root of the superior mesenteric vein (station 14v) in advanced gastric cancer remains controversial. Although station 14v is not within the routine scope of D2 lymphadenectomy in the 3rd edition of the Japanese Gastric Cancer Treatment Guidelines, it has been observed that D2+ station 14v lymph node dissection may improve overall survival (OS) in clinically staged III/IV patients with middle- and lower-third gastric cancer [54]. The Expert Committee recommends these indications for the dissection of station 14v lymph nodes: clinically staged III patients with tumors located at the middle and lower parts of the stomach, and having lymph node metastasis, especially for those with metastasis to the infra-pyloric lymph nodes.
- Although it has been reported that preventive para-aortic lymph node dissection cannot improve the long-term survival of patients with resectable advanced gastric cancer [55], the value of therapeutic para-aortic lymph node dissection is still controversial. Suitable patients should be encouraged to participate in clinical trials.

*Laparoscopic surgery*

- For distal gastrectomy of gastric cancer classified as cT1N0 and cT1N1, laparoscopic surgery is equivalent to open surgery in terms of safety and short-term prognosis [56]. Therefore, laparoscopic surgery is recommended as a routine surgical technique.
- Till present, there is no large-scale prospective study regarding laparoscopic total gastrectomy for early-stage gastric cancer. Although there is not sufficient scientific-based evidence, the Expert Committee suggests that it can be performed in experienced medical centers.
- For advanced gastric cancer, small-scale studies and large-scale retrospective studies have reported that laparoscopic surgery had an advantage over open surgery in regards to short-term efficacy, but their long-term efficacy was equivalent [57, 58]. Preliminary results of an ongoing phase III clinical study have shown that laparoscopic distal gastrectomy combined with D2 lymphadenectomy was safe [59], but the long-term survival is yet to be published. The Expert Committee recommends that laparoscopic surgery for advanced gastric cancer should be carried out in large experienced medical centers, where standardized technical facilities and safety can be guaranteed.

*Digestive tract reconstruction*

- The type of digestive tract reconstruction performed is to depend on the patient’s physical condition and the surgeon’s experience as far as it does not affect the radicality of the gastrectomy.
- Billroth I and Billroth II surgeries are mostly adopted for distal gastrectomy. For tumors located in the lower third of the stomach, especially those invading the pylorus and the duodenum, Billroth II surgery is recommended because these patients can have a second chance for surgery in case of tumor recurrence [60]. Roux-en-Y anastomosis can effectively reduce bile reflux and prevent the occurrence of remnant gastritis. However, this operation is relatively complex, and the risk of postoperative retention syndrome may be increased [61].
- Gastroesophageal anastomosis is frequently used for proximal gastrectomy, but the risk of esophageal reflux is common and serious. Modified tubular gastroesophageal anastomosis can significantly reduce the risk of severe esophageal reflux. Compared with gastroesophageal anastomosis, the Jejunal interposition method can reduce the occurrence of moderate or severe esophageal reflux, but this operation is complicated and the risks for frequent abdominal discomfort, upper abdominal fullness, and hiccups are common [62]. Therefore, its advantages remain to be confirmed [63]. If required, it is suggested that the Jejunal interposition method should be carried out in large experienced medical centers.
- Roux-en-Y is the preferred reconstruction method for total gastrectomy. It has been reported that, in addition to Roux-en-Y anastomosis, the reconstruction of the Jejunal pouch digestive tract may improve the patients’ postoperative quality of life [61]. The Jejunal interposition method as a replacement for the stomach is complicated, and there is still controversy concerning its efficacy in improving the patients’ quality of life. If required, it is suggested that this procedure should be carried out in large experienced medical centers.

*3.1.1.2.3 Perioperative treatment of resectable gastric cancer*

*3.1.1.2.3.1 Adjuvant treatment*

**Table:** 

Treatment method	Stratification*	Grade I recommendations	Grade II recommendations	Grade III recommendations
Postoperative adjuvant treatment	pT3–4NanyM0 pTanyN + M0 R0 D2 resection	Postoperative adjuvant chemotherapy: XELOX (Evidence 1A) S-1 alone (Evidence 1A)	Postoperative adjuvant chemotherapy: FOLFOX (Evidence 2A) SOX (Evidence 2A)	Postoperative adjuvant chemotherapy: XP (Evidence 2B) Postoperative adjuvant chemoradiotherapy: DT 45–50.4 Gy (concurrent fluoropyrimidine) (Evidence 3)
	pT2-4NanyM0, R0 resection; Failing to meet criteria for D2	Postoperative chemoradiotherapy: DT 45–50.4 Gy (concurrent fluoropyrimidine) (Evidence 1A)	MDT discussion for optimal treatment regimen	
	pT2-4NanyM0 R1/R2 resection	Postoperative chemoradiotherapy: DT 45 to 50.4 Gy (concurrent fluoropyrimidine)	MDT discussion for optimal treatment regimen	

*XELOX* oxaliplatin (xeloda) + capecitabine, *FOLFOX* leucovorin calcium (folinic acid) + fluorouracil + oxaliplatin, *SOX* S-1 + oxaliplatin, *XP* capecitabine + cisplatin

* According to the 8th AJCC/UICC pathological staging system (pTNM) for gastric cancer

*3.1.1.2.3.2 Neoadjuvant therapy*

**Table:** 

Treatment method	Stratification*	Grade I recommendations	Grade II recommendations	Grade III recommendations
Neoadjuvant therapy	cT3-4aN + M0, stage cIII		Neoadjuvant therapy: FOLFOX (Evidence 2A) PF (Evidence 2A) XELOX (Evidence 2A) SOX (Evidence 2A) FLOT (Evidence 2A) Postoperative adjuvant therapy after R0 resection to continue the preoperative drug regimen**	Neoadjuvant therapy: ECF (Evidence 2B) mECF (Evidence 2B) After R0 resection, to continue the preoperative drug regimen as postoperative adjuvant therapy**
	cT3-4aN + M0, stage cIII: EGJ carcinoma	Neoadjuvant chemoradiotherapy: DT 45–50.4 Gy (concurrent fluoropyrimidine, platinum or taxanes) (Evidence 1B)	Neoadjuvant chemotherapy (same regimen as above) (Evidence 2A) Neoadjuvant radiotherapy (for patients intolerant of chemotherapy) (Evidence 2B)	
	cT4bNanyM0, stage cIVA (no unresectable factors)	MDT discussion for an optimal treatment regimen	Participation in clinical trials is encouraged	
	Disease progression after neoadjuvant therapy	MDT discussion for an optimal treatment regimen	Participation in clinical trials is encouraged	
	R1/R2 resection after neoadjuvant therapy	MDT discussion for an optimal treatment regimen	Participation in clinical trials is encouraged	

*FOLFOX* leucovorin calcium (folinic acid) + fluorouracil + oxaliplatin, *PF* cisplatin + 5-fluorouracil (5-FU), *FLOT* 5-FU + leucovorin + oxaliplatin + docetaxel, *ECF* epirubicin + cisplatin + 5-FU, *mECF* modified ECF

* According to the 8th AJCC/UICC clinical staging system (cTNM) for gastric cancer

** For patients who were preoperatively assessed by radiological/pathological examination as having a positive response to neoadjuvant therapy

**Notes**

*Adjuvant treatment for resectable gastric cancer*

- Adjuvant chemotherapy is indicated for preoperative non-treated patients who had D2 radical gastrectomy and pathologically diagnosed as T2–4 and (or) N+. Their recommended regimen is capecitabine combined with oxaliplatin or cisplatin [64], or S-1 alone [65]. There is not sufficient scientific evidence to support the use of adjuvant chemotherapy for stage I (T1N1M0 and T2N0M0) patients. The Expert Committee recommends adjuvant chemotherapy for patients with lymph node metastasis. Adjuvant chemotherapy may reduce the risk of metastasis in the following groups of patients: high-risk T2N0 patients, younger age (< 40 years old), high histological grade or poorly differentiated lesions, and those with nervous, vascular, or lymphatic invasion.
- Phase III clinical trials on adjuvant chemoradiotherapy for resectable gastric cancer in Eastern and Western countries demonstrated different results [66, 67]. The Chinese Expert panel [40] recommends that patients with resectable gastric cancer failing to meet the D2 radical gastrectomy criteria and those with a high risk of local recurrence (high lymph node metastasis rate, inadequate resection margin, etc.) should be treated with 5-fluorouracil (5-FU)-based regimen or capecitabine in combination with cisplatin followed by concurrent chemoradiotherapy.
- For locally advanced gastric cancer patients who fail to meet the R0 resection criteria, postoperative chemoradiotherapy is recommended [68] or an MDT discussion should be performed to decide the optimal treatment.

*Preoperative and perioperative chemotherapy for advanced gastric cancer*

- Perioperative therapy (neoadjuvant chemotherapy/radiotherapy + surgery + adjuvant chemotherapy/radiotherapy) for gastric cancer has been proven to be superior to surgery alone in Western countries [69, 70]. Preoperative chemotherapy prior to radical gastrectomy in Asian countries has also demonstrated significantly improved tumor remission rates and R0 resection rates with safer profiles [71, 72]. The survival benefits of perioperative chemo-/radiotherapy as compared with postoperative chemotherapy for radical D2 gastrectomy remains to be determined with large phase III clinical trials.
- The present recommendations for preoperative chemotherapy include epirubicin + cisplatin + 5-FU (ECF) [69], cisplatin + 5-FU (PF) [73], modified ECF (mECF) [74], oxaliplatin + capecitabine (XELOX) [75], oxaliplatin + 5-FU (FOLFOX) [76], and oxaliplatin + S-1 (SOX) [77]. Based on the data of the FLOT4-AIO study presented at the 2017 American Society of Clinical Oncology (ASCO) annual meeting [78], the FLOT regimen (docetaxel combined with oxaliplatin and 5-FU/leucovorin [LV]) demonstrated a prolonged median disease-free survival and median OS, higher pathological response rate and R0 resection rate with a more tolerable profile as compared to the ECF/ECX (Epirubicin + Cisplatin + Capecitabine) regimen. Therefore, the FLOT regimen can be considered as the new standard preoperative chemotherapy regimen for resectable gastric cancer.
- Neoadjuvant chemoradiotherapy + surgery + adjuvant chemotherapy was proven successful in the clinical studies of EGJ adenocarcinoma. However, scientific-based evidence for its therapeutic benefits on tumors at other primary locations within the stomach, especially as compared with perioperative chemotherapy, is inadequate and requires further confirmation from phase III clinical trials. The long-term follow-up results of the POET study showed that preoperative chemoradiotherapy could reduce local recurrence and tended to prolong survival as compared with preoperative chemotherapy [79]. Also, results from the RTOG-9904 multi-center phase II clinical trial demonstrated satisfactory results for locally advanced gastric cancer patients undergoing preoperative chemoradiotherapy [80]. Therefore, the current recommendation for stage III EGJ carcinoma is neoadjuvant chemoradiotherapy followed by radical D2 gastrectomy. Preoperative chemoradiotherapy for locally advanced gastric cancer should be carried out in clinical trials. Recommended regimens for concurrent chemotherapy include paclitaxel combined with 5-FU, paclitaxel combined with platinum, or 5-FU combined with platinum. At present, the TOPGEAR clinical trial and a multicenter phase III prospective clinical trial launched by the Sun Yat-sen University Cancer Center (NCT01815853) are actively investigating the effects of preoperative chemoradiation therapy in this category of patients [81].
- The efficacy of neoadjuvant therapy should be timely evaluated using these following recommended imaging modalities: EUS, CT, or PET/CT.
- Compared with CT and other non-invasive imaging examinations, laparoscopic exploration can improve the diagnostic rates of occult metastasis within the abdominal cavity, including radiologically undetected small liver metastases. It can be carried out alongside a cytological examination of intraperitoneal washings. Explorative laparoscopic staging is recommended prior to prescribing neoadjuvant therapy.
- For surgically resected specimens diagnosed as pathological complete response (pCR) after neoadjuvant therapy, it is recommended that the same neoadjuvant regimen to be continued postoperatively as the adjuvant regimen. Till present, there is no sufficient evidence attributing to the survival differences between those who undergo different adjuvant regimens as to their initial neoadjuvant regimens or abstain from adjuvant therapies.
- In case of disease progression following neoadjuvant therapy, surgery should be considered if R0 resection can be achieved. If not, the following treatment should be decided through an MDT discussion.
- For patients who could not have R0 gastrectomy despite the absence of distant metastasis after neoadjuvant chemotherapy, either postoperative chemoradiotherapy is recommended or the following treatment should be decided by an MDT discussion. If chemoradiotherapy is performed prior to the surgery, the following treatment should be decided by an MDT discussion or else palliative treatment is recommended.

#### Comprehensive treatment of unresectable gastric cancer

**Table:** 

Staging	Stratification	Grade I recommendations	Grade II recommendations	Grade III recommendations
Unresectable	ECOG performance score = 0–1	Concurrent chemoradiotherapy (Evidence 1A)^a,c^ MDT should discuss the possibility of surgery after concurrent chemoradiotherapy. If complete resection can be achieved, surgery can be considered	Chemotherapy (Evidence 2B)^b^ Radiotherapy (Evidence 2B)^c^ MDT should discuss the possibility of surgery after chemotherapy or radiotherapy. If complete resection can be achieved, surgery can be considered	Chemotherapy^b^ + radiotherapy or concurrent chemoradiotherapy^a,c^ MDT should discuss the possibility of surgery after sequential chemotherapy or concurrent chemoradiotherapy. If complete resection can be achieved, surgery can be considered
	ECOG performance score = 2	Best supportive care or symptomatic treatment (Evidence 1A) Bypass surgery, endoscopic treatment, stenting, and/or palliative radiotherapy are recommended if they may improve nutritional status, alleviate bleeding, pain, or obstruction	Best supportive care or symptomatic treatment + chemotherapy ± radiotherapy (Evidence 2A) After improvement of nutritional, symptomatic status and depending on the patients’ general conditions, chemotherapy^b^ alone or in combination with radiotherapy can be considered	

*ECOG* Eastern Cooperative Oncology Group

^a^Concurrent chemoradiotherapy regimen: Chemotherapy regimen: capecitabine + paclitaxel [82] (Evidence level 1A); cisplatin + 5-FU or capecitabine or S-1 [83] (Evidence level 1A); oxaliplatin + 5-FU or capecitabine or S-1 [84] (Evidence level 2B); paclitaxel + 5-FU or capecitabine or S-1 [80] (Evidence level 2B); capecitabine [66] (Evidence level 2B); S-1 [85] (Evidence level 2B); 5-FU [86] (Evidence level 1A)

^b^For more details regarding the chemotherapeutic regimens, please refer to “late-stage metastatic gastric cancer chemotherapy regimen”

^c^Radiotherapy: Three-dimensional conformal radiotherapy/intensity-modulated radiotherapy

**Notes**

- Gastric adenocarcinomas are considered unresectable if (1) the primary tumor shows extensive invasion and cannot be separated from the surrounding normal tissues or has encased major vascular structures; (2) regional lymph node metastases are fixed and fused in groups and/or are not within the scope of surgical resection; (3) the patient shows contraindications to surgery or refuses surgical intervention due to poor general condition, malnutrition, severe hypoproteinemia, severe anemia, or severe underlying diseases. if they are: (1) associated with these tumor-related factors: (a) extensive invasion of the primary tumor which cannot be separated from the surrounding normal tissues or has encased major vascular structures; (b) regional lymph node metastases are fixed and fused in groups and/or are not within the scope of surgical resection; (2) contraindicated to surgery or refusal for surgical intervention due to poor general condition, malnutrition, severe hypoproteinemia, severe anemia, severe underlying diseases.
- Combined examination modalities are recommended for accurate clinical stage evaluation and for judging the resectability of the tumor. Special consideration should be made regarding peritoneal metastasis/dissemination as they are common, particularly in advanced stage (T3–4 or N+) patients, and laparoscopic exploration with a cytological examination of the intraperitoneal washings is recommended prior to treatment initiation.
- Concurrent chemoradiotherapy is recommended for patients with unresectable locally advanced gastric cancer who have a good general condition. Studies have shown that, in terms of tumor downstaging and pathological remission, concurrent chemoradiotherapy was superior to chemotherapy or radiotherapy alone, and the survival time of these patients could be prolonged if a local control of the tumor could be achieved. For those patients who had a favorable response to radiotherapy, the tumor should be re-evaluated to judge the potential for surgical resectability. Further, there have been reported studies demonstrating survival benefits for locally advanced patients with a good general condition who had radical or even palliative resection [87, 88].
- For patients who are unsuitable for concurrent chemoradiotherapy due to extensive tumor or lymph nodes invasion, whereby wide irradiation fields may cause more harm than benefits, chemotherapy or radiotherapy alone can be considered as an alternative [89]. For patients with favorable responses, an MDT discussion is recommended after the treatment to judge the potential for surgical resection. If the tumor is still unresectable, sequential or concurrent chemoradiotherapy may be considered, and tumor resectability should be re-evaluated after the treatment.
- Radiologists should perform a comprehensive evaluation based on the patients’ physical condition and the scope of the irradiation field before performing sequential or concurrent chemoradiotherapy. In general, concurrent chemoradiotherapy is superior to radiotherapy alone [90]. Radiotherapy alone should be used when patients cannot tolerate concurrent chemoradiotherapy. However, patients who had prior chemotherapy may have poor tolerance to radiotherapy, and dual-drug regimen combined with concurrent chemoradiotherapy may reduce the completion rate of radiotherapy. In this situation, single-drug 5-FU regimen with concurrent chemoradiotherapy can be considered.
- Appropriate radiotherapy planning can be considered. For patients with potentially resectable disease, in addition to the visible tumors (primary, metastatic tumors or lymph nodes) confirmed by imaging examinations, appropriate external expansion of the irradiation field can be considered to include regions of lymphatic drainage. For unresectable patients, the irradiation field should comprise of visually identified tumors only, and preventive irradiation of lymph nodes should not be considered. The recommended dosage for preoperative radiotherapy is DT 40–45 Gy. After treatment, the tumor should be re-assessed to judge whether the patient can undergo surgery or continue radiotherapy. The recommended total dose of radical radiotherapy is DT 50–60 Gy. The recommended dose for palliative radiotherapy is DT 30–40 Gy. As a note, the dosage and scope of irradiation should be based on the patient’s general condition, the size of the irradiation field, expected lifespan, and possible irradiation damage to normal tissues and organs.
- For patients who can tolerate chemotherapy, it has been observed that, as compared with best supportive care, chemotherapy can prolong the survival of metastatic gastric cancer patients [91]. As such, for patients presenting with severe gastrointestinal obstruction, hemorrhage, or obstructive jaundice at first diagnosis, it is suggested that nutrition tube, stent implantation, gastrointestinal bypass surgery, local palliative radiotherapy, acid inhibition, hemostasis, and analgesia should be prescribed, preferentially within the first 2–4 weeks of presentation, as longer duration may result in tumor progression. Chemotherapy can be considered when the patient’ general condition improves. If not, best supportive care can be continued. The main chemotherapy drug regimen includes 5-fluorouracil-based, platinum-based, taxanes-based, irinotecan regimen. Combined chemotherapy can result in a response rate of 30%–54% and a median OS of 8–13 months [91]. Although combined chemotherapy is more effective than single-drug chemotherapy, 5-FU alone can still be considered for those patients who cannot tolerate combined chemotherapy [92].
- Radiotherapy can significantly alleviate some clinical symptoms of late-stage gastric cancer patients, such as hemorrhage, severe cancer pain, dysphagia, and obstruction and can improve the patients’ general condition and quality of life [93]. Palliative radiotherapy may be considered for those patients with old age, advanced disease, decreased cardio-pulmonary functions, multiple underlying diseases, and difficulty to sustain surgical intervention.
- Three-dimensional conformal radiotherapy (3D-CRT) and intensity-modulated radiotherapy (IMRT) are recommended as related studies have demonstrated that, compared with conventional two-dimensional radiotherapy, 3D-CRT or IMRT was excellent at targeting the dose distribution area and at protecting normal organ tissue, especially in the gastrointestinal tract, liver, and kidneys, against adverse events from irradiation [94, 95].

### Treatment of metastatic gastric cancer

For the patients who cannot undergo radical resection or with metastatic/recurrent disease, comprehensive treatment based on systemic antitumor therapy is recommended. Other therapeutics such as palliative surgery, radiotherapy, radiofrequency ablation, intraperitoneal perfusion, and arterial embolization may help to prolong survival and improve quality of life. Therefore, we must emphasize that treatments for such patients should be discussed by an MDT to assess the optimal personalized treatment strategy.

At present, drugs for gastric cancer include mainly chemotherapeutic and molecular targeted drugs for which their applicability is supported by sufficient scientific-based evidence and experiences in clinical practice. Programmed cell death protein 1 (PD-1) monoclonal antibody has already been approved by the US Food and Drug Administration (FDA) and in Japan as the third-line treatment for advanced gastric cancer, but in China, its approval by the CFDA is yet to be given. Accordingly, these patients are encouraged to participate in immunotherapy-based clinical trials. The treatment of metastatic gastric cancer is challenging, especially since the available second- and third-line regimens are limited and are yet to demonstrate significant efficacies. There are still no effective molecular targeted drugs for the first-line treatment of HER2-negative patients, and these patients should be encouraged to participate in clinical trials. In addition, the stomach is an important digestive organ where the primary lesion may directly affect the nutritional status, leading to complications such as bleeding, digestive tract obstruction, and/or perforation. Therefore, maintenance of nutritional status, as well as active prevention and timely treatment of complications, should be given special attention during the entire antitumor treatment process.

#### Choice of antitumor drug treatment for late-stage metastatic gastric cancer

*First-line treatment*

**Table:** 

HER2 status	Grade I recommendations	Grade II recommendations	Grade III recommendations
Positive	Trastuzumab in combination with fluoropyrimidine/capecitabine + cisplatin (Evidence 1A)	Trastuzumab in combination with other first-line chemotherapy regimens (e.g., oxaliplatin + capecitabine or S-1 + cisplatin) (Evidence 2B)	Trastuzumab in combination with other first-line chemotherapy regimens excluding anthracyclines (Evidence 3)
Negative	Cisplatin + fluoropyrimidine (5-FU/capecitabine/S-1) (Evidence 1A)	Three-drug combination regimens (e.g., DCF and mDCF) may be suitable for patients in good physical conditions and with large tumor burden (Evidence 2A)	Three-drug combination regimens (e.g., ECF and mECF) may be suitable for patients in good physical conditions and with large tumor burden (Evidence 2A)
	Oxaliplatin + fluoropyrimidine (5-FU/capecitabine/S-1) (Evidence 2B)		
	Docetaxel + 5-FU/capecitabine/S-1 (Evidence 2B)	Single-drug regimens (e.g., fluoropyrimidine- or taxanes-based therapy) may be suitable for those in poor physical condition (Evidence 2B)	Irinotecan-based chemotherapy (Evidence 3)
	Paclitaxel + 5-FU/capecitabine/S-1 (Evidence 2B)		

*ECF* epirubicin + cisplatin + 5-FU, *DCF* docetaxel + cisplatin + 5-FU, *mDCF* modified DCF

*Second-line treatment*

**Table:** 

HER2 status	ECOG score	Grade I recommendations	Grade II recommendations	Grade III recommendations
Positive	0–1	Encourage participation in clinical trials	If platinum therapy fails and trastuzumab has not been used, trastuzumab in combination with paclitaxel is suggested (Evidence 1A/2A)	If trastuzumab has not been used, trastuzumab in combination with a second-line chemotherapy regimen (excluding anthracyclines) is suggested. Refer to the second-line options for HER2-negative gastric cancer (Evidence 3)
	2	Encourage participation in clinical trials		
Negative	0–1	Mono-chemotherapy (docetaxel or paclitaxel or irinotecan) (Evidence 1)	Dual-drug chemotherapy with paclitaxel- or fluoropyrimidine-based regimen (Evidence 2B)	If there is no history of treatment failure with platinum, cisplatin- or oxaliplatin-based chemotherapy is suggested (Evidence 3)
		Encourage participation in clinical trials		
	2	Paclitaxel alone (Evidence 1A)		
		Encourage participation in clinical trials		

*Third-line treatment (both HER2-positive and -negative)*

**Table:** 

ECOG score	Grade I recommendations	Grade II recommendations	Grade III recommendations
0–1	Apatinib (Evidence 1A)	Mono-chemotherapy (Evidence 3)	PD-1 monoclonal antibody (Evidence 1A)
	Encourage participation in clinical trials		
2	Encourage participation in clinical trials	Best supportive care	Mono-chemotherapy (Evidence 3)

**Notes**

- Difference in antitumor treatment for late-stage gastric cancer may arise due to heterogeneity in ethnicity and tumor location. Therefore, these patients should be encouraged to participate in clinical trials.
- Fluoropyrimidine, platinum, and taxanes are the main therapeutic drugs for late-stage gastric cancer. Usually, first-line regimens are based on fluoropyrimidine combined with platinum and/or taxanes to constitute a two- or three-drug regimen. Adequate clinical evidence is available to support the recommendation of fluoropyrimidine combined with platinum. Fluoropyrimidine combined with taxanes have also demonstrated adequate efficacy and safety in clinical studies [75, 96–101]. In China, the two-drug therapy consisting of fluoropyrimidine and platinum is recommended, and the selection of first-line chemotherapy regimens should be based on the patients’ physical condition, age, and any underlying disease.
- There is no sufficient evidence to recommend chemotherapeutic drugs based on the prediction of chemotherapeutic response according to the Lauren classification, molecular classification, in vitro drug susceptibility test, xenograft transplantation model, xenobiotic metabolism, or metabolomics. Patients suspected of fluoropyrimidine metabolic disorders are advised to undergo a dihydropyrimidine dehydrogenase deficiency (DPD) test.
- The standard treatment for late-stage gastric cancer usually lasts 4–6 months, and these patients should be regularly followed-up after disease control. Although there is no large-scale clinical study to demonstrate the OS benefit of maintenance treatment with sequential monotherapy after standard chemotherapy over standard chemotherapy alone, a preliminary study has shown that maintenance therapy could improve the patients’ quality of life by decreasing adverse events [102].
- Peritoneal metastasis is the most common type of metastasis observed in late-stage gastric cancer patients and is considered as a leading cause of death. For those with symptomatic ascites, drainage and hyperthermic intraperitoneal perfusion chemotherapy (HIPEC) can be considered. For patients with asymptomatic ascites, first-, second-, or third-line chemotherapeutic regimens can be used. The Phoenix-GC study compared HIPEC plus intravenous paclitaxel and S-1 with standard SP regimen (intravenous infusion of cisplatin combined with oral S-1) in patients with peritoneal metastasis as a first-line therapy [103]. Survival benefits were observed in the subgroup with moderate amount of ascites. However, the median OS of the entire studied population was not prolonged (17.7 vs. 15.2 months, *P* = 0.080). As a result, HIPEC is not recommended for routine use in clinical practice.
- Most patients who were given second-line chemotherapy had an ECOG score of 0–1, and few had a score of 2 when they were enrolled in phase III clinical trials. Therefore, the risks and benefits of second-line treatments for the patients in poor physical condition should be carefully considered.
- Regarding the second-line chemotherapy, at present, there are monotherapy regimens that have been recommended based on the results from phase III clinical trials [102, 104]. In some small-scale phase II clinical trials, it was observed that dual-drug chemotherapy for the patients with an ECOG score of 0–1 had better tumor control with an acceptable toxicity profile as compared with the observational arm. As such, combination chemotherapy can be considered for patients in good physical condition if the risks and benefits of the treatment are fully weighed-up.
- Clinical studies regarding the third-line treatment for advanced gastric cancer involved a limited number of patients. As such, the benefit of chemotherapy remains to be further clarified. In clinical practice, it is emphasized that the risks and benefits of treatment should be carefully weighed-up depending on the patients’ physical condition, underlying diseases, tumor-related symptoms, and risk of complications. It is suggested that monotherapy should be prioritized.
- The ToGA trial showed that, compared with chemotherapy alone, trastuzumab in combination with first-line chemotherapy improved the efficacy and survival in HER2-overexpressed late-stage gastric cancer patients [23]. Many phase II clinical studies have demonstrated the efficacy and safety of trastuzumab in combination with other different chemotherapy regimens. For HER2-positive metastatic gastric cancer patients with first-line chemotherapy failure and who had no prior treatment with trastuzumab, data from a phase II clinical study have demonstrated treatment efficacy and safety for using paclitaxel in combination with trastuzumab [105]. However, if trastuzumab was used in the first-line therapy, there is no high-level evidence to suggest its cross-line application. Preliminary results of a multi-center prospective observational study from China have shown that continued application of trastuzumab in the second-line therapy could prolong the median progression-free survival (PFS) [106]. The Chinese Society of Clinical Oncology, the Chinese Anti-Cancer Association Gastric Cancer Specialized Committee, and the Chinese Anti-Cancer Association Tumor Pathology Specialized Committee jointly led the publication of the “consensus of Chinese experts on molecular targeted treatment of HER2-positive advanced gastric cancer”, which can help oncologists to improve the diagnosis and treatment of HER2-positive gastric cancer [22].
- HER2-targeted drugs, such as anti-HER2 monoclonal antibody pertuzumab, small-molecule tyrosine kinase inhibitor lapatinib [107, 108], drug-coupled anti-HER2 monoclonal antibody TDM-1 [109], did not show positive results in phase III clinical trials and are not recommended for clinical use.
- Anti-angiogenic drugs include anti-VEGF antibody (bevacizumab), anti-VEGFR antibody (ramucirumab), and small-molecule tyrosine kinase inhibitor (regorafenib, apatinib, and so on). The REGARD study showed that compared with placebo, ramucirumab alone as a second-line drug could prolong the survival of metastatic gastric cancer patients with tolerable adverse events [110]. The RAINBOW study showed that compared with paclitaxel alone, second-line ramucirumab combined with paclitaxel could prolong survival even further [111], which led to the approval of ramucirumab by the US FDA as a second-line treatment for late-stage gastric cancer. A phase III clinical study which enrolled 273 patients who had treatment failure after using second-line/subsequent-lines chemotherapeutic regimens showed that apatinib, compared with the placebo, could prolong the median progression-free survival (mPFS) (2.6 vs. 1.8 months, *P* < 0.001) and increase the disease control rate (42.05% vs. 8.79%, *P* < 0.001) [112]. Accordingly, on October 10, 2014, the CFDA approved the use of apatinib, a highly selective VEGFR-2 inhibitor, as a third-line/above therapy for late-stage gastric cancer patients. In the *Clinical Application of Apatinib* [113], the CSCO Experts panel provides clinicians with references regarding the application and safety of apatinib [113].
- The use of immunological checkpoint inhibitors has been supported by prospective studies. Based on the results of the ONO-4538-12 study [114], nivolumab was approved as a third-line treatment for advanced gastric cancer in Japan. In contrast with placebo, nivolumab significantly reduced the risk of death by 37%, and the observed 1-year survival rate was also higher in the nivolumab arm (26.2%) than in the placebo arm (10.9%). Based on the results of the KEYNOTE-059 study [115], the US FDA approved pembrolizumab as a third-line drug for the treatment of metastatic gastric/EGJ adenocarcinoma expressing PD-L1 level ≥ 1%. In one study, when 259 gastric cancer patients who were refractory to previous treatments were given pembrolizumab as a single agent, they demonstrated satisfactory mPFS, mOS, and ORR of 2 months, 6 months, and 12%, respectively (PD-L1 positive rate, 16%) [115]. In 2017, the US FDA approved pembrolizumab for MSI-H or dMMR solid tumors as a third-line treatment. However, the KEYNOTE-061 study showed that pembrolizumab, as a second-line therapy, did not prolong OS or mPFS in PD-L1-positive patients compared with standard chemotherapy [116]. As such, there is still controversy concerning the actual benefit from immunologic checkpoint inhibitors [117].
- The risk of malnutrition is high in gastric cancer. The guidelines from the China Anti-cancer Association for Cancer Nutrition and Support Therapy [118] recommend that the nutritional status of patients with gastric cancer should be screened and evaluated. Patients with a moderate or severe malnutritional risk should undergo appropriate nutritional therapy alongside chemotherapy, and when necessary, chemotherapy should be delayed [118].

#### Comprehensive treatment of recurrent or metastatic gastric cancer with solitary distant metastasis

A solitary distant metastatic lesion is defined as one that has the possibility of being locally treated, regardless of the primary gastric lesion and regional lymph nodes [119–121].

There are no large-scale prospective randomized controlled clinical study data to provide scientific-based evidence for the treatment of gastric cancer with solitary distant metastasis. Most of the evidences are from retrospective or small-scale studies. Therefore, the optimal therapeutic option for such patients should be discussed through an MDT, and the patients should be encouraged to participate in clinical trials.

##### Treatment of gastric cancer with postoperative local recurrence or solitary distant metastasis

**Table:** 

Site	Stratification	Grade I recommendations	Grade II recommendations
Local recurrence	ECOG performance score = 0–1, no concomitant disease, had no perioperative radiotherapy	Comprehensive treatment based on systemic antitumor drug therapy or encourage to participate in clinical trials	Surgery in combination with drug therapy^a^ (Evidence 2B) Radiotherapy combined with drug therapy^b^ (Evidence 2A)
	ECOG performance score ≥ 2, serious concomitant disease, or history of perioperative radiotherapy		
Solitary distant liver metastasis	ECOG performance score = 0–1, no concomitant disease		Local treatment (surgery or radiofrequency therapy) combined with drug therapy^c^ (Evidence 2A)
	ECOG performance score ≥ 2, or serious concomitant disease		
Ovarian metastasis	ECOG performance score = 0–1, no concomitant disease, lateral or bilateral ovarian metastasis		Ovariectomy combined with drug therapy^d^ (Evidence 2A)
	ECOG performance score ≥ 2 or serious concomitant disease		

**Notes**

- Local recurrence is defined as the re-occurrence of tumor at the resection site after radical gastrectomy. Most studies regarding local recurrence of gastric cancer were retrospective and came from single institutions, and there is a lack of large-scale prospective study. Preliminary results suggest that surgery may be an important prognostic factor as the mOS of these surgically operated patients reached 25.8 months, while those non-surgical patients were only 6.0 months [122]. Although some local recurrent diseases can be surgically treated, the indications for surgical intervention must be strictly followed.
- Patients with local recurrence who did not receive any previous radiotherapy may gain great greater survival benefits from concurrent chemoradiotherapy. A retrospective study identified that gastric cancer patients with local recurrence at the anastomotic site who underwent concurrent chemoradiotherapy achieved an ORR of 61.9% and mOS of 35 months. Compared with chemotherapy alone, concurrent chemoradiotherapy resulted in a higher ORR (87.8% vs. 63.0%, *P* = 0.01), longer mOS (13.4 months vs. 5.4 months, *P* = 0.06), and better control of symptoms such as pain, bleeding, and obstruction (85.0% vs. 55.9%, *P* = 0.006) [123].
- Liver metastasis that occurs more than 6 months after radical gastrectomy is described as metachronous liver metastasis. Solitary liver metastasis is defined as liver metastasis confined to a single lobe, only one lesion being ≤ 4 cm in diameter, and has no involvement with the surrounding blood vessels and bile ducts. There are few clinical studies on gastric cancer patients with solitary liver metastasis after gastrectomy. Both the results of retrospective studies and meta-analysis have shown that the survival of patients undergoing liver surgery was longer than those patients not receiving liver surgery (mOS: 22–26 months vs. 3–7 months, respectively, *P* < 0.001). However, there were no survival differences observed between patients with metachronous and synchronous liver metastasis [124, 125]. Radiofrequency ablation (RFA) is a local treatment for solitary liver metastasis. Retrospective studies have shown that, compared with systemic chemotherapy, RFA could significantly prolong the mOS of patients with metachronous liver metastasis (25 months vs. 12 months, *P *= 0.015) [126]. Literature has also identified that patients receiving RFA combined with systemic chemotherapy could achieve an mPFS of 9.8 months and an mOS of 20.9 months [127]. A multi-center retrospective study in Japan has shown that the patients with solitary or multiple liver metastases could have an mOS of up to 3.40 years and an mRFS of up to 0.98 years after surgical resection and/or local treatment [128]. The survival difference between surgical resection and non-surgical local treatment was not significant, but the patients with N0/N1 disease could benefit significantly from surgery or local treatment. Therefore, surgery and/or local treatment may be an effective treatment for patients with solitary liver metastasis after gastric cancer surgery. However, it is not clear whether postoperative systemic chemotherapy should be given.
- Ovarian resection combined with antitumor drug therapy is important for female gastric cancer patients with postoperative metachronous ovarian metastasis. It has been shown that, compared with systemic chemotherapy alone, ovarian resection combined with chemotherapy could significantly prolong mOS (9 months vs. 19 months) [129]. The surgical benefits for patients with metachronous ovarian metastasis may be superior to those with simultaneous ovarian metastasis (mOS, 36 months vs.17 months) [130].

##### Treatment of stage IV gastric cancer with single distant metastasis at initial diagnosis

**Table:** 

Site	Stratification	Grade I recommendations	Grade II recommendations	Grade III recommendations
Peritoneal cytology positive (P0CY1)*	ECOG performance score = 0–1, radical resection of the primary lesion and regional lymph nodes	To be treated as recurrent/metastatic gastric cancer or encourage to participate in clinical trials	Standard D2 gastrectomy followed by postoperative chemotherapy^b^ (Evidence 2B) Conversion chemotherapy combined with radical surgery^c^ (Evidence 2B)	
Retroperitoneal lymph node metastasis (station No. 16a2/b1)	ECOG performance score = 0–1, radical resection of the primary lesion and regional lymph nodes		Conversion chemotherapy combined with radical surgery^d^ (Evidence 2B)	Radical surgery combined with chemoradiotherapy (Evidence 3)
Single liver metastasis	ECOG performance score = 0–1, radical resection of the primary lesion and regional lymph nodes		Systemic chemotherapy combined with local treatment^e^ (Evidence 2A)	Surgical resection of primary and metastatic tumor combined with systemic chemotherapy^f^ (Evidence 2B)
Ovarian metastasis	ECOG performance score = 0–1, radical resection of the primary lesion and regional lymph nodes			Surgery combined with systemic chemotherapy^g^ (Evidence 2B)

* P0CY1: No peritoneal metastasis, but the peritoneal cytology is positive for carcinoma cells

**Notes**

- Gastric cancer patients with peritoneal metastasis can be classified into the following categories: (1) patient with carcinoma cells detected by peritoneal cytology, without visible metastatic lesions (P0CY1); and (2) patient with macroscopic metastatic lesions in the abdominal cavity which are not considered as single distant metastatic lesion [131].
- Scientific-based data regarding the treatment of gastric cancer patients with positive exfoliative cytology are not enough, and there is a need for large-scale, prospective randomized control trials. The results of CCOG0301 study demonstrated that CY1 patients could benefit from S-1 as adjuvant chemotherapy after radical gastrectomy [132], and it was further reported that their mOS could reach up to 22.3 months [133].
- Selected P0CY1 gastric cancer patients may benefit from systemic chemotherapy in combination with surgery. However, there are no definitive conclusions about the timing, indications, and surgical methods for the operation. CY1 gastric cancer patients may benefit from preoperative chemotherapy. The results of relevant studies suggest that patients with good preoperative therapeutic responses may have a chance to undergo radical surgery, following which their median survival could be extended from 12.6 to up to 43.2 months [134]. The efficacy of preoperative chemotherapy and lymph node involvement are important factors affecting their OS [135, 136]. Another study has reported that CY1 patients treated with HIPEC (paclitaxel used as intraperitoneal perfusion) combined with S-1 and paclitaxel as systemic chemotherapy, providing that their exfoliative cytology changed to negative and they could undergo radical surgery, their OS was observed to increase from 14.3 (without surgery) to 30.5 months (with radical surgery) [137]. Intraoperative peritoneal chemotherapy (IPC) and intraoperative extensive peritoneal lavage (EIPL)-IPC are also other recommended options. Findings from a meta-analysis have shown that the combination of surgery with IPC could increase the 5-year survival rate (risk ratio [RR] = 3.10) of late-stage gastric cancer patients and reduce their risk of recurrence (odds ratio [OR] = 0.45); if IPC was combined with EIPL, this benefit could be further increased (RR = 6.19, OR = 0.13) [138]. Overall, some highly selected gastric cancer patients with P0CY1 disease can benefit from surgery combined with intraoperative chemotherapy or systemic chemotherapy. However, the appropriate timing, indications, and surgical procedures for such patients after conversion chemotherapy are still unclear.
- Till present, there is no large-scale prospective randomized controlled clinical trial for the treatment of solitary distant metastasis to the para-aortic lymph nodes (station no. 16a2/b1) in gastric cancer patients. The results from a retrospective study have shown that patients with good performance score, single retroperitoneal lymph node metastasis, and good response to chemotherapy could obtain a survival benefit if their primary tumor could be resected (R0 resection) and could have a 3-year survival rate reaching up to 40% [139]. Results from the JCOG0405 study showed that the 5-year survival rate for gastric cancer patients with solitary para-aortic lymph node metastasis was 57% if they had undergone 2 cycles of neoadjuvant chemotherapy using S-1 and cisplatin followed by subsequent D2 gastrectomy with para-aortic lymph node dissection [140]. Observations from the JCOG1002 study [141] have shown that the addition of docetaxel to S-1 plus cisplatin regimen resulted in a clinical remission rate of 57.7%, R0 resection rate of 84.6%, and pathological remission rate of 50.0%, but no data for the 5-year survival rate was provided. As such, this double-drug regimen (S-1 + cisplatin) is still considered as the first choice of treatment for this group of patients. A prospective study conducted by the Zhongshan Hospital Affiliated to Fudan University demonstrated that the PFS of patients with solitary para-aortic lymph node metastasis after neoadjuvant chemotherapy and radical surgery could reach up to 18.1 months [142]. In the REGATTA study, the subgroup analysis of lymph node metastasis, as the only incurable factor, also demonstrated good efficacy of surgical resection [143]. Data from larger-scale clinical trials are needed to confirm the role of surgery in patients with para-aortic lymph node metastasis as the only incurable factor.
- The data from randomized controlled trials on patients with solitary liver metastasis is still lacking. Some retrospective studies and meta-analysis have confirmed that for some highly selected patients with solitary liver metastasis treated with systemic chemotherapy in combination with R0 resection of the primary and metastatic lesions, greater survival benefits could be observed [144–146]. However, there are no definite conclusions about the timing, indications, and surgical methods. For patients unsuitable for surgery, clinicians should carefully assess other local treatments in combination with systemic therapies. It is highly recommended that treatment decisions are made through an MDT discussion.
- There is no large-scale, prospective, randomized controlled clinical trial regarding the treatment for gastric cancer patients with solitary distant liver metastasis. Some retrospective studies have confirmed that after strict screening, for selective patients, survival benefits could be gained through radical resection of the primary lesion and metastatic lesion versus palliative resection of the primary tumor only [147]. A systematic review showed that the 5-year survival rate for patients undergoing radical resection of the primary tumor and liver metastasis could reach up to 23.8%, with a median survival time of 22 months [148]. A systematic review of 39 retrospective studies found that the resection of liver metastasis could significantly improve prognosis (hazard ratio [HR] = 0.50; *P* < 0.001), especially for those with solitary liver metastasis and in the Eastern population [125]. Another meta-analysis also demonstrated that the prognosis of patients undergoing hepatectomy for metastatic liver lesion was significantly better than that of those who did not undergo surgery, with a median survival of 23.7 months vs. 7.6 months [125]. In 2017, the EORTC and JCOG conducted a questionnaire survey in 17 European countries and 55 research centers in Japan and found that, regarding gastric cancer patients with liver metastasis whose primary and metastatic lesions could be removed, most centers recommend these patients to receive preoperative chemotherapy followed by primary and metastatic resection [149]. Retrospective studies have shown that T1–3 disease, H1, R0 resection followed by systemic chemotherapy were important prognostic factors for gastric cancer patients with liver metastasis [150, 151]. In addition, it has been found that the HER2 gene amplification rate in gastric cancer patients with liver metastasis was higher and that these patients would be more likely to gain a survival benefit from anti-HER2 targeted therapy [152].
- Krukenberg tumors are the metastatic lesion of gastric cancer that have been metastasized to the ovary (one or both). For gastric cancer patients with single-ovary metastasis, systemic chemotherapy is regarded as the main treatment. Findings from some retrospective studies performed on these patients have confirmed that systemic chemotherapy combined with surgical resection of the primary tumor and/or ovarian metastasis could provide some survival benefits, extending their mOS from 6–9 months to 19–23.7 months [153]. Their most determining prognostic factors were an ECOG performance score of 0–1, R0 resection (radical resection of the primary lesion and the ovarian metastatic lesion), and postoperative systemic chemotherapy [129]. Some highly selected patients with single-ovarian metastasis may benefit from surgery combined with systemic chemotherapy. However, the selection of patients, timing of treatments, and methods for such operations are still unclear.

## Follow-up visits

**Table:** 

Purpose^a,b^	Grade I recommendations	Grade II recommendations
Follow-up visits for early-stage gastric cancer patients after radical gastrectomy	Once every 6 months in the first 3 years, followed by once a year until 5 years after surgery	Once every year for more than 5 years after surgery
	Follow-up contents*: Clinical history, physical examination, blood chemistry (including CEA and CA19-9), HP detection, performance status monitoring, weight monitoring, annual chest, abdominal, and pelvic CT scan or ultrasound (especially for those with abnormal CEA levels)	1. Chest, abdominal, and pelvic enhanced CT 2. PET/CT, MRI 3. Gastroscopy (recommended once a year)^d,e^
Follow-up visits^c^ for advanced gastric cancer patients after radical resection, or non-resectable gastric cancer patients after palliative treatment	Once every 3 months in the first 2 years, followed by once every 6 months until 5 years	Once every year for more than 5 years after treatment
	Follow-up contents: Clinical history, physical examination, blood chemistry (including CEA and CA19-9), HP detection, performance status monitoring, weight monitoring, chest, abdominal, and pelvic CT every 6 months (especially for those with abnormal CEA levels)	1. Chest, abdominal, and pelvic enhanced CT 2. PET/CT, MRI 3. Gastroscopy (recommended once a year)^d,e^
New symptoms or symptom deterioration	Follow-up visit at any time	

* The follow-ups are to be performed once a year unless specified otherwise

**Notes**

- The main objective of follow-up/monitoring is for the timely identification and intervention for the plausibility of radical treatment in gastric cancer patients with local recurrence, metastasis, or secondary recurrence after showing a satisfactory response to the treatment administered, with the aim to improve their OS and quality of life [154]. Large-scale evidence-based data supporting the best follow-up visit/monitoring strategy is still lacking.
- Planification of follow-up visits should be made on an individualized basis relating the specific conditions of the patient [155]. Routine tumor follow-up/monitoring is not recommended for patients with poor physical conditions and is unsuitable for antitumor treatment in the event of recurrence.
- HP infection has been found to have a direct implication on the prognosis of gastric cancer patients. It is recommended that HP detection should be performed as a routine examination in follow-up visits.
- The postoperative follow-up visits for advanced gastric cancer patients who have undergone radical resection is similar, irrespective whether or not they have had neoadjuvant chemo-/radiotherapy [19].
- The main purpose of follow-up gastroscopy after gastrectomy is to assess the status of the anastomoses and to timely identify any abnormalities. Local recurrence at the anastomotic site is rare. However, for any abnormalities observed, an adequately sized local biopsy should be performed.
- The follow-up strategies of gastroscopy are as follow: (1) gastroscopy is recommended within 1 year after surgery; (2) patients are advised to undergo annual follow-up gastroscopy; (3) if there is evidence of high-grade atypical hyperplasia or signs of gastric cancer recurrence, adequately sized biopsy should be made, and a follow-up gastroscopy should be performed within 1 year if no cancerous tissue was observed [41].
- PET/CT and MRI are currently not recommended as routine follow-up/monitoring imaging modalities. They are only recommended for suspected recurrence when there is no clear evidence from conventional imaging examinations (CT or ultrasound) despite a continuous elevation of blood tumor markers (e.g., CEA and CA19-9).

## Appendix

### References

#### Categories of evidence from the 2018 CSCO clinical practice guidelines for common malignant tumors

**Table:**

Level of evidence			CSCO expert consensus
Category	Quality of level	Source	
1A	High	Based on data from well-structured and rigorously controlled meta-analysis, and/or large-scale, randomized controlled clinical trials	Uniform consensus reached (support level: ≥ 80%)
1B	High	Based on data from well-structured and rigorously controlled meta-analysis, and/or large-scale, randomized controlled clinical trials	Consensus reached with minimum disagreement (support level: 60%–80%)
2A	Relatively low	Based on data from meta-analysis, small-scale, randomized controlled trials, well-designed large-scale retrospective studies, and/or case–control studies	Uniform consensus reached (support level: ≥ 80%)
2B	Relatively low	Based on data from meta-analysis, small-scale, randomized controlled trials, well-designed large-scale retrospective studies, and/or case–control studies	Consensus reached with minimum disagreement (support level: 60%–80%)
3	Low	Based on data from single-arm clinical studies, case reports, and/or expert opinions	No consensus reached and has major disagreement (support level: < 60%)

#### Recommendation grades from the 2018 CSCO clinical practice guidelines for common malignant tumors

**Table:**

Recommendation grade	Criteria
Grade I	Evidence level 1A and some Evidence level 2A: Grade I recommendations include Evidence level 1A and some Evidence level 2A which obtained high consensus from the expert panel and has suitable applicability for Chinese gastric cancer patients Specifically, in the CSCO Guidelines, Grade I recommendations include the following: universally accepted measures with clear indications for diagnosis and treatment, has adequate applicability for Chinese gastric cancer patients, and is included in the National Reimbursement Drug List (NRDL). The priority for allocating Grade I recommendations is solely for the benefits of the patients and is independent to changes regarding commercial medical insurance
Grade II	Evidence level 1B and some Evidence level 2A: Grade II recommendations include Evidence level 1B and some Evidence level 2A which obtained satisfactory consensus with minimum disagreements from the expert panel and has limited applicability for Chinese gastric cancer patients Specifically, Grade II recommendations include the following: high-level evidence provided by multi-center studies that have been randomly controlled internationally or domestically (in China), but may have limited applicability for Chinese patients or low potency ratio, in addition to drugs or treatments that may exceed the purchasing power of the general public of gastric cancer patients; treatments that are expensive but may have substantial benefits for the patients are also regarded as Grade II recommendations
Grade III	Evidence level 2B and 3: Despite the lack of strong evidence-based data, however, these are recommendations that have obtained satisfactory consensus with minimum disagreements from the expert panel and are provided as a reference for medical personnel usage
Not recommended/objection	Recommendations for which the expert panel has uniform consensus that there is adequate evidence to prove that the drugs or medical technologies do not have sufficient benefits or may even cause harm to Chinese patients. These are labeled as “experts do not recommend” or, when applicable as “experts’ disapproval”. It can be allocated to any grade recommendations
